# Protein kinase inhibitor SU6668 attenuates positive regulation of Gli proteins in cancer and multipotent progenitor cells

**DOI:** 10.1016/j.bbamcr.2014.01.003

**Published:** 2014-04

**Authors:** Alla Piirsoo, Lagle Kasak, Mari-Liis Kauts, Mart Loog, Kairit Tints, Piia Uusen, Toomas Neuman, Marko Piirsoo

**Affiliations:** a Protobios LLC, Mäealuse 4, Tallinn 12618, Estonia; b Department of Gene Technology, Tallinn University of Technology, Akadeemia tee 15, Tallinn 12618, Estonia; c Institute of Technology, University of Tartu, Nooruse 1, 50411 Tartu, Estonia; d Cellin Technologies LLC, Mäealuse 4, Tallinn 12618, Estonia

**Keywords:** AP, alkaline phosphatase, ASCs, adipose tissue derived stromal cells, GliACT, transcriptional activator form of Gli proteins, GliFL, full-length Gli proteins, Ptch, patched, RNAi, RNA interference, Shh, Sonic Hedgehog, Smo, Smoothened, shRNA, short hairpin RNA, siRNA, small interfering RNA, Sufu, Suppressor of Fused, TGF-β, Transforming Growth Factor β, Ulk3, unc-51-like kinase 3, WB, Western blot, HSC, Hedgehog signaling complex, qRT-PCR, quantitative real-time PCR, Gli proteins, Signal transduction, Differentiation, Multipotent cells, Inhibitor

## Abstract

Observations that Glioma-associated transcription factors Gli1 and Gli2 (Gli1/2), executers of the Sonic Hedgehog (Shh) signaling pathway and targets of the Transforming Growth Factor β (TGF-β) signaling axis, are involved in numerous developmental and pathological processes unveil them as attractive pharmaceutical targets. Unc-51-like serine/threonine kinase Ulk3 has been suggested to play kinase activity dependent and independent roles in the control of Gli proteins in the context of the Shh signaling pathway. This study aimed at investigating whether the mechanism of generation of Gli1/2 transcriptional activators has similarities regardless of the signaling cascade evoking their activation. We also elucidate further the role of Ulk3 kinase in regulation of Gli1/2 proteins and examine SU6668 as an inhibitor of Ulk3 catalytic activity and a compound targeting Gli1/2 proteins in different cell-based experimental models. Here we demonstrate that Ulk3 is required not only for maintenance of basal levels of Gli1/2 proteins but also for TGF-β or Shh dependent activation of endogenous Gli1/2 proteins in human adipose tissue derived multipotent stromal cells (ASCs) and mouse immortalized progenitor cells, respectively. We show that cultured ASCs possess the functional Shh signaling axis and differentiate towards osteoblasts in response to Shh. Also, we demonstrate that similarly to *Ulk3* RNAi, SU6668 prevents *de novo* expression of Gli1/2 proteins and antagonizes the Gli-dependent activation of the gene expression programs induced by either Shh or TGF-β. Our data suggest SU6668 as an efficient inhibitor of Ulk3 kinase allowing manipulation of the Gli-dependent transcriptional outcome.

## Highlights

• Ulk3 is involved in the maintenance of Gli1/2 expression.
• SU6668 prevents *de novo* expression of Gli1/2 proteins induced by Shh or TGF-β.
• SU6668 inhibits up-regulation of Gli1/2 proteins *via* Ulk3.
• Human ASCs differentiate towards osteoblasts in response to Shh.

## 1 Introduction

Misregulation of cellular signaling pathways, that are important in embryonic development and maintaining adult homeostasis, leads to inherited as well as sporadic diseases. One of such pathways, where a clear correlation between abnormal pathway activation and disease progression has been observed, is the Sonic Hedgehog (Shh) signaling pathway [1]. Disruption or misregulation of the Shh pathway results in various developmental abnormalities including holoprosencephaly, Pallister–Hall syndrome, Gorlin syndrome, Greig cephalopolysyndactyly, Rubinstein–Taybi syndrome and different types of cancer (basal cell carcinoma, medulloblastoma, glioma, breast, pancreatic, prostate cancers and more). Similarly important is the TGF-β signaling pathway, with its role in various types of cancer, vascular diseases and fibrosis [2], [3].

The Shh pathway utilizes Gli proteins (Gli1, 2, 3) as transcriptional effectors. According to the widely accepted paradigm, differentiated regulation of Gli proteins occurs in an Hh signal dependent way. In the absence of the ligand, *Gli1* is transcriptionally repressed; full-length Gli2 and Gli3 (Gli2/3FL) proteins are bound by a putative cytoplasmic complex called Hedgehog signaling complex (HSC). HSC may consist of a number of proteins including Suppressor of Fused (Sufu), kinesin-like protein Kif7, unc-51-like kinase 3 (Ulk3), and Gli2/3FL transcription factors [4], [5], [6], [7], [8]. Gli2/3FL proteins bound by HSC are phosphorylated for degradation and processing into the transcriptional repressor forms (Gli2/3REP) [9], [10], [11], [12]. Activation of Shh pathway leads to rapid stabilization and activation of Gli2/3FL probably through yet uncharacterized phosphorylation events, their relocation to the nucleus and up-regulation of their target genes, for instance *Ptch1* and self-amplifying *Gli1* [7], [12]. *Gli2* has been also suggested as a transcriptional target of Shh signaling in mouse CNS during embryonic development [13]. Although both proteins, Gli2 and Gli3, may be involved in primary mediation of Shh activities, the role of Gli2 activator is more crucial, whereas Gli3 acts mainly as a transcriptional repressor [14], [15], [16].

Gli proteins are known to be regulated independently of Hh ligands on both transcriptional and post-translational levels. Mouse Gli1 protein can be activated *via* Erk1/2 kinases, and *Gli2* is shown to be up-regulated in the epidermis of mice over-expressing TGF-β1 [17], [18]. Also, the TGF-β1/SMAD3/TCF4/β-catenin signaling axis controls human *GLI2*, and consequently *GLI1*, expression [18], [19]. Regulation of Gli2 in bone metastases and tumor-induced osteolysis also occurs independently of the canonical Shh pathway [20].

Most of the small molecule inhibitors of the Shh pathway identified so far target trans-membrane SMO oncoprotein responsible for triggering the intracellular signaling cascade following the ligand binding to another trans-membrane protein PTCH1. In addition, several inhibitors of GLI proteins and Shh itself have been identified (reviewed in Ref. [21]). However, no inhibitors targeting the activity of either HSC complex or protein kinases required for activation of GLI proteins have been reported. The latter might be effective not only in Shh pathway inhibition, but also in alleviating TGF-β/GLI dependent signaling events.

SU6668 ((Z)-5-[(1,2-dihydro-2-oxo-3H-indol-3-ylidene)methyl]-2,4-dimethyl-1H-pyrrole-3-propanoic acid; TSU68) has been shown to inhibit several tyrosine and serine/threonine protein kinases in an ATP competitive manner [22], [23]. The affinity chromatography experiment using a resin covalently bound with SU6668 has revealed that additionally to the previously known targets, SU6668 is capable to bind a number of other protein kinases including ULK3 [23]. We have recently identified Ulk3 as an important Gli regulator. However, a mechanism of regulation of the *Ulk3* gene and possible interrelations between endogenous Ulk3 and Gli proteins remains unclear.

Adipose tissue derived stromal cells (ASCs, also known as mesenchymal stem or progenitor cells) have been extensively investigated during the last decade. These heterogeneous cell populations have evoked a great interest for regenerative medicine due to their non-immunogenic phenotype and capacity to respond to appropriate inducers by increasing expression of markers specific for different mesodermal lineages, such as adipocytes, chondrocytes or osteoblasts [24], [25], [26]. The Shh signaling pathway has not been extensively characterized in human ASCs, although one research group has reported that activation of Shh signaling negatively regulates differentiation of ASCs towards osteoblasts triggered by osteogenic cocktail [27]. However, these studies were conducted using Shh-conditional media or SMO agonists added to ASCs in the presence of osteogenic inductors, whereas influence of Shh itself on native ASCs has not been analyzed. In contrast, the osteogenic capacity of Shh in mouse ASCs and C3H10T1/2 is well documented [28], [29]. Differentiation of osteoprogenitors occurs under control of Runx2, a factor essential for bone formation and skeletal development [30], [31]. *Runx2* is expressed from two alternative promoters at least in two isoforms. Both *Runx2* isoforms are expressed in osteoblasts and participate in differentiation [30], [32]. Osteogenesis is characterized by expression of lineage-specific proteins, such as early markers Sp7 and alkaline phosphatase (AP) and late markers osteopontin (Opn) and osteocalcin (Bglap) [29], [33], [34]. Gli2/3 proteins as mediators of Hh activities participate not only in positive regulation of osteogenesis but also in early chondrogenesis in mice [35], [36], [37], whereas adipogenesis is inhibited by activation of the Shh signaling [28], [38]. Expression and activities of GLI1/2 proteins in human ASC tri-lineage differentiation programs have not been described.

The current study aims to investigate whether the mechanism of activation of Gli1 and Gli2 (Gli1/2) proteins has similarities regardless of signaling pathway evoking that. In answering this question, we examine SU6668 as a small molecule inhibitor able to prevent activation of Gli1/2 proteins in both Shh and TGF-β signaling pathways in an Ulk3 dependent manner. Finally, we provide novel data in the field of stem cell biology relating to possible roles of Shh signaling and GLI1/2 proteins in ASC differentiation programs.

## 2 Materials and methods

### 2.1 Ethic statement

Donors of the primary cells provided written informed consent to participate in this study in accordance with the approval for research with human materials No 159 from 14th of February, 2013 by Ethics Committee of National Institute for Health Development, Tallinn, Estonia.

### 2.2 Proteins and chemicals

FLAG-tagged ULK3 and GLI2, Shh and His-tagged ULK3-Ubi proteins were purified as previously described [6], [39], [40]. SU6668 was dissolved in DMSO (both from Sigma-Aldrich, Steinheim, Germany) and stored at − 70 °C prior use. Human recombinant TGF-β1 and TGF-β3 were purchased from PeproTech (Rock Hill, NJ, USA). Human insulin, dexamethasone (DEX), IBMX, indomethacin and ascorbate-2-phosphate were purchased from Sigma-Aldrich, while ITS supplement was purchased from Gibco, Invitrogen (Carlsbad, CA, USA).

### 2.3 Cell culture

Peripheral blood mononuclear cells (PBMCs) and human ASCs were isolated and characterized as previously described [41]. The donors of primary cells are described in Supplementary Table 1. Freshly isolated PBMCs were frozen in D-MEM containing 1 g/L glucose (Gibco), supplemented with 50% of Fetal Bovine Serum (FBS) (PAA, Pasching, Austria) inactivated at 56 °C for 30 min (HI-FBS) and 10% DMSO and stored in liquid nitrogen prior to use. The C3H10T1/2 cell line was a generous gift from Prof. Rune Toftgård's lab (Centre for Nutrition and Toxicology, Karolinska Institute, Sweden). The MDA-MB-231 and 3T3-L1 cell lines were purchased from ATCC. MDA-MB-231 and 3T3-L1 cells were propagated in D-MEM containing 4.5 g/L glucose (Gibco) supplemented with 10% FBS and 1% penicillin/streptomycin mix (PEST) (Gibco). C3H10T1/2 and ASCs were propagated in D-MEM containing 1 g/L glucose supplemented with 10% of HI-FBS and 1% PEST. The cells were grown at 37 °C and 5% CO_2_. All treatments of the cells except inductions of ASCs towards chondrocytes were conducted in the respective base growth medium supplemented with 3% of FBS or HI-FBS, 1% PEST, 12 nM Shh or 10 ng/ml of TGF-β3 and different concentrations of SU6668, if indicated. Adipogenic differentiation was induced by 10 μg/ml of human insulin, 1 μM dexamethasone, 0.5 mM IBMX and 10 μM indomethacin. Chondrogenic differentiation was conducted in DMEM-high glucose containing 10 ng/ml of TGF-β1, 1×  ITS supplement, 100 μM ascorbate-2-phosphate, 1 μM DEX and 1% PEST. Media were replenished every 2 d.

### 2.4 Alkaline phosphatase activity

C2H10T1/2 cells were washed with PBS and lysed in Lysis Solution (Tropix, Bedford MA, USA). Alkaline phosphatase (AP) activity was measured using CSPD substrate with Sapphire-II™ Enhancer (Invitrogen) and Genios Pro combined fluoro-and luminometer (Tecan Group Ltd., Männedorf, Switzerland). Total protein concentrations were measured using BCA Protein Assay kit (Pierce Biotechnology Inc., Rockford, IL, USA) and used for normalization of AP activity values.

### 2.5 Over-expression studies

Synthetic Negative siRNA (Silencer Negative Control 2, Neg. siRNA), Silencer® Select siRNAs S89965 and S89966 against mouse *Ulk3* and S24886 and S24887 against human *ULK3* were purchased from Ambion (Austin, TX, USA). Synthetic siRNAs were delivered to cells using Lipofectamine RNAiMax reagent (Invitrogen). 3T3-L1 cells were transfected with 20 nM of siRNAs. Initial reverse and 24 h later forward transfection procedures were conducted using the same amounts of siRNAs and transfection reagent. ASCs were transfected with 10 nM siRNAs using forward transfection protocol. The cells were treated with DMSO, SU6668 or/and 12 nM Shh or 10 ng/ml TGF/β1 24 h after forward transfection. MDA-MB-231 cells were transfected with constructs encoding human FLAG-tagged GLI2, mouse GFP-tagged Gli2, pcDNA3.1 or pmax-GFP (Lonza Inc.) using Lipofectamine 2000 (Invitrogen) [6], [40]. To measure transfection efficiency, the cells were fixed in 4% PFA for 30 min, washed with PBS and analyzed using Accuri C6 flow cytometer.

### 2.6 Western blot

**Supplementary Fig. 1:**
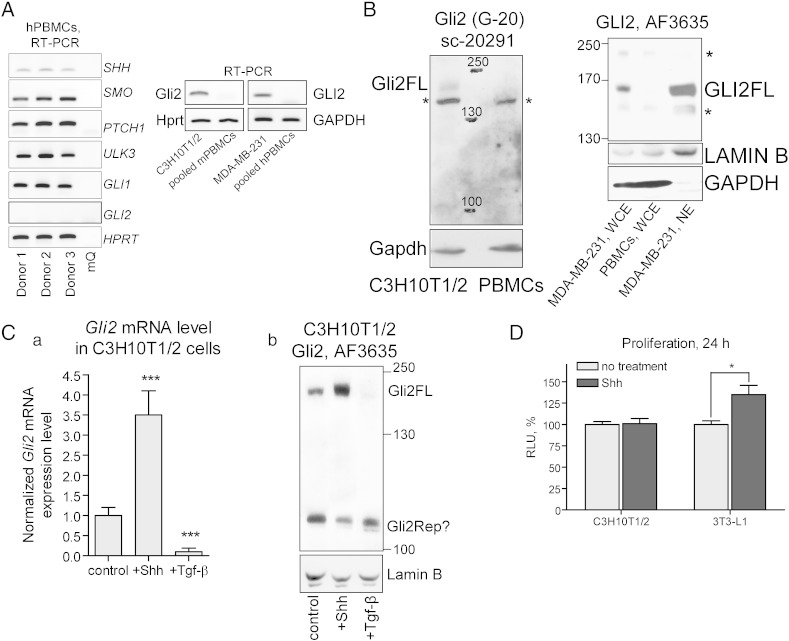
(A) Expression of *Gli2* was analyzed in C3H10T1/2, MDA-MB-231 and peripheral blood mononuclear cells (PBMCs) of mouse or human origin using RT-PCR. Expression of *SHH*, *SMO*, *PTCH1*, *ULK3* and *GLI1* was detected using RT-PCR in PBMCs isolated from three different donors. (B) Mouse Gli2 and human GLI2 proteins were analyzed in C3H10T1/2, PBMCs and MDA-MB-231 cell lysates by immuno-blotting using GN2 antibodies G-20 (sc-20291, Santa Cruz Biotechnology) and AF3635 (R&D Systems). Approximately 60 μg of total protein of whole cell extracts (WCE) or 70 μg of nuclear extract (NE) were loaded per line; Gli2FL — full-length Gli2, * — unspecific bands. (C) C3H10T1/2 cell were treated with Shh or Tgf-βi for 48 h. Expression levels of *Gli2* mRNA and protein were analyzed using RT-PCR and WB; *** — p < 0.001; Gli2Rep? — putative repressor form of Gli2. (D) C3H10T1/2 and 3 T3-L1 cells were treated with Shh for 24 h. Proliferation of the cells were tested using ViaLightTM plus kit and normalized with amount of total protein. The data are presented as an average mean of three independent experiments ± S.D., * — p < 0.05.

Western blot (WB) was performed as previously described [41]. WB analyses were done using 40–60 μg of total protein per lane. All immuno-blotting assays were performed at least in three replicates and representative images are shown. Commercially available antibodies and their dilutions are listed in Supplementary Table 2. Gli2 AF3635 antibody has been described previously [7]. The specificity of Gli2 antibodies was tested and representative images are shown in Supplementary Fig. 1A–C. Runx2 antibody was generated in Labas Ltd. (Tartu, Estonia) against a peptide STLSKKSQAGASELG and used in dilution 1:2000. WB images were quantified using ImageQuant software.

**Fig. 1:**
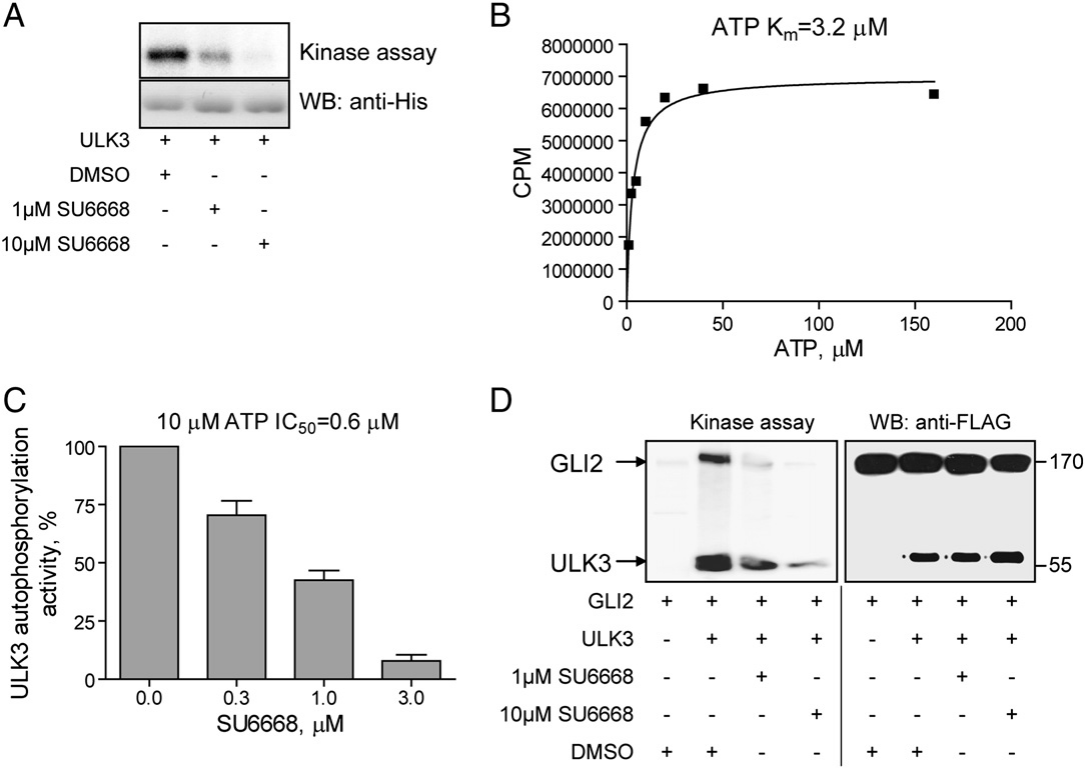
SU6668 inhibits ULK3 catalytic activity *in vitro*. (A) Bacterially expressed and purified His-tagged ULK3 protein was subjected to *in vitro* kinase assay in the presence of 2 μM ATP and vehicle or SU6668. The presence of ULK3 protein was verified by WB. (B) His-tagged ULK3 protein was subjected to *in vitro* kinase assay in the presence of indicated concentrations of ATP. The Michaelis–Menten curve was built using levels of ULK3 autophosphorylation activity normalized with amount of ULK3 protein identified by Coomassie staining (both quantified using ImageQuant software). (C) ULK3 protein was subjected to *in vitro* kinase assay in the presence of 10 μM ATP. Different concentrations of SU6668 were included into each reaction. The intensity of ULK3 autophosphorylation and the amount of ULK3 protein identified by Coomassie staining were quantified using ImageQuant software. The normalized level of ULK3 autophosphorylation activity in the presence of vehicle was set as 100%. (D) GLI2 and ULK3 were over-expressed and immuno-purified using a resin conjugated with FLAG antibody. The purified proteins were mixed and subjected to *in vitro* kinase assay (left panel). The proteins were verified by WB (right panel).

### 2.7 *In vitro* kinase assay and statistical analysis

*In vitro* kinase assay and statistical analysis were performed as described [6]. IC_50_, *K*_m_ and *K*_i_ values were calculated using GraphPad Prism software.

### 2.8 Immunocytochemistry

Primary cilia and nuclei of ASCs were stained as previously described [42] using antibodies against acetylated tubulin (Sigma-Aldrich), Alexa Fluor 568 goat anti-mouse IgG (Invitrogen), and Mounting Medium with DAPI (Abcam). The cells were visualized using fluorescent microscope Olympus BX61 with UPLan SApo 40 × objective.

### 2.9 Nuclear extract preparation

Nuclear and cytoplasmic extracts were prepared using NE-PER® Nuclear and Cytoplasmic Extraction Reagents (Thermo Scientific). Approximately 40 μg of the extracts was used for WB analysis.

### 2.10 Quantitative real time PCR

Quantitative real time PCR (qPCR) was performed as described [41]. The data of qPCR analyses are expressed as the normalized with *Hprt* or *GAPDH* average means ± S.D. of three measurements obtained from at least three independent experiments. Expression of genes demonstrating Ct values above 37 were considered as undetectable. The primers used are listed in Supplementary Table 3.

## 3 Results

### 3.1 SU6668 inhibits ULK3 kinase activity *in vitro*

ULK3 is an active serine/threonine kinase able to phosphorylate itself and GLI proteins [40]. It has been previously shown that a tyrosine kinase inhibitor SU6668 can physically interact with ULK3 in a chromatographic pull-down assay [23]. In order to test, whether SU6668 is able to inhibit ULK3 kinase activity, bacterially expressed and purified His-tagged ULK3 protein was tested in the *in vitro* kinase assay in the presence of 1 or 10 μM SU6668. SU6668 inhibited ULK3 autophosphorylation activity in a concentration dependent manner (Fig. 1A).

In order to determine the *K*_i_ of SU6668 for ULK3, first the *K*_m_ of ATP towards bacterially purified ULK3 was measured and found to be 3.2 μM under these conditions (Fig. 1B). Next, increasing concentrations of SU6668 were used in the presence of 10 μM ATP in the *in vitro* kinase assay (Fig. 1C). Under these conditions, SU6668 was able to inhibit the ULK3 autophosphorylation activity with an IC_50_ of 0.6 μM. The calculated *K*_i_ of SU6668 for ULK3 was found to be approximately 0.2 μM.

In order to exclude tag-specific phenomena and verify the effect of SU6668 using other ULK3 substrates, over-expressed and immuno-purified FLAG-tagged ULK3 and GLI2 proteins were subjected to *in vitro* kinase assay in the presence of 2 μM ATP and SU6668. Phosphorylation of GLI2 by ULK3 was completely inhibited by 10 μM SU6668; however, some residual catalytic activity of ULK3 was detectable in the presence of 1 μM SU6668 (Fig. 1D, left panel). The presence of the proteins tested in the *in vitro* kinase assay was confirmed by WB (Fig. 1D, right panel). These data demonstrate that SU6668 is an efficient inhibitor of ULK3 kinase in the *in vitro* kinase assay.

### 3.2 SU6668 inhibits the Shh signal transduction

Since Ulk3 is able to modulate the activity of Gli proteins and to interact with SU6668, the effects of SU6668 on the Shh pathway execution was tested in two Shh-responsive mouse cell lines C3H10T1/2 and 3T3-L1. Shh stimulates differentiation of C3H10T1/2 toward osteoblasts without significant increase in proliferation [29], [33]. In contrast, pre-adipocytes 3T3-L1 demonstrated increased proliferation rate in response to Shh (Supplementary Fig. 1D).

**Fig. 2:**
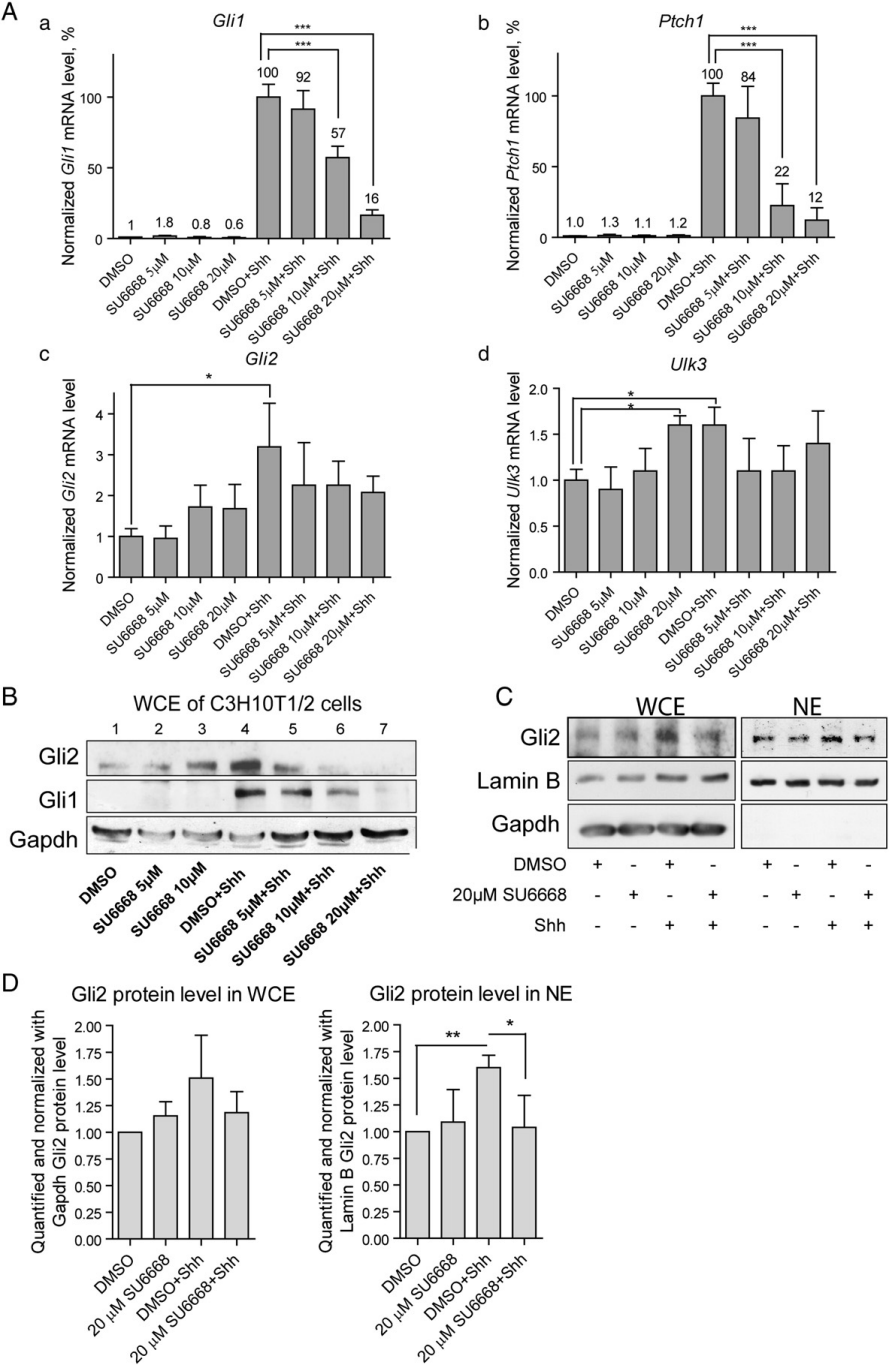
SU6668 hinders Shh signaling in C3H10T1/2 cells. (A, B) C3H10T1/2 cells were treated as indicated for 42 h. The levels of expression of *Gli1*, *Ptch1*, *Gli2* and *Ulk3* were analyzed by qPCR and normalized by the level of *Hprt* mRNA expression. The normalized level of the respective gene mRNA in the control sample treated with DMSO was set as 1. The data are presented as an average mean ± S.D.; *—p < 0.05, ***—p < 0.001, n = 4. (C) C3H10T1/2 cells were treated as indicated for 2 h. The cells were split for whole cell extract and nuclear extract preparation (WCE and NE, respectively). The proteins were analyzed by WB. Gli2 was detected using Gli2 G-20 (B) or AF3635 (C) antibodies. (D) Gli2, Lamin B and/or GAPDH images were quantified. Gli2 protein levels were normalized with Lamin B and/or GAPDH levels and set as 1 in the control cells treated with DMSO. Data are presented as an average mean ± S.D.; *—p < 0.05, **—p < 0.01, n = 3.

**Supplementary Fig. 2:**
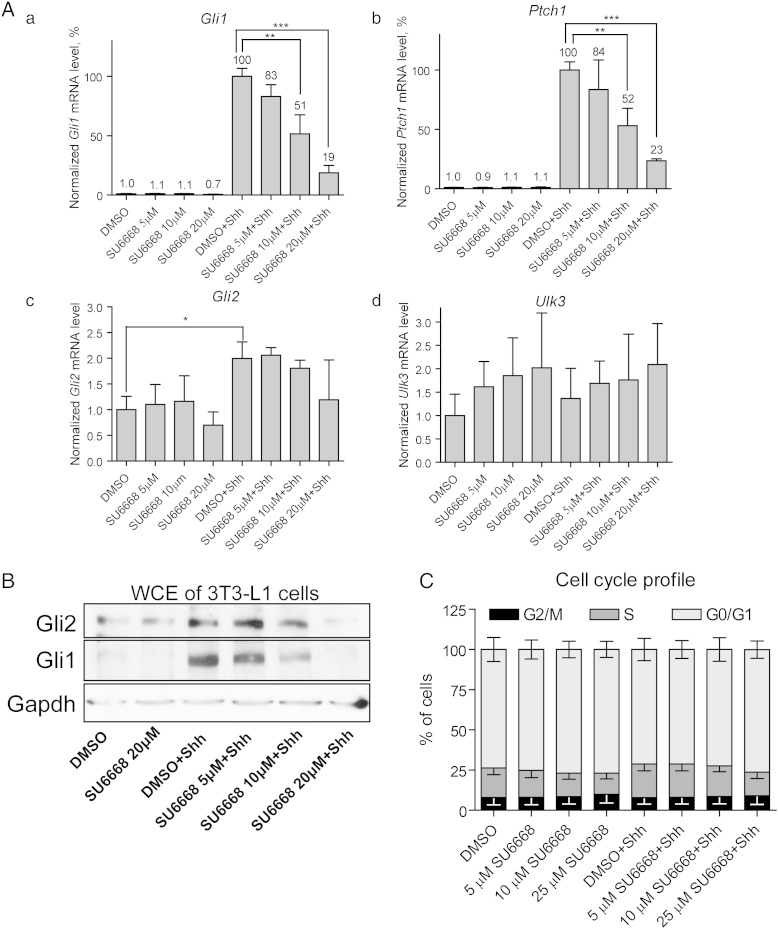
(A, B) 3 T3-L1 cells were treated as indicated for 30 h, trypsinized and split for total RNA and protein isolation. (A) Expression levels of *GIH*, *Ptchl*, *Gli2* and *Ulk3* were analyzed by qPCR and normalized with the level of *Hprt* expression. The level of the respective gene mRNA expression in the control sample was set as 1, and the data from other samples were calculated relative to 1. The level of *Gli1* and *Ptch1* expressions in the samples treated with Shh and DMSO was set as 100%. *Gli1* and *Ptch1* expression levels in the samples treated Shh and SU6668 were calculated relative to 100%. The data are presented as an average mean ± S.D.; *—p < 0.05, **—p < 0.01, ***p < 0.001; n = 4. (B) Gli1, Gli2 and GAPDH proteins were analyzed by immuno-blotting. Approximately 60 μg of total protein of whole cell extracts (WCE) was loaded per line. (C) C3H10T1/2 cells were treated as indicated for 24 h. Cell cycle profile was analyzed using BrdU staining and BD FACSCalibur flow cytometer.

C3H10T1/2 and 3T3-L1 cells were treated with Shh and/or 5, 10 or 20 μM SU6668 for 42 h. Expression levels of *Gli1*, *Ptch1*, *Gli2* and *Ulk3* were analyzed by qPCR (Fig. 2 and Supplementary Fig. 2, for C3H10T1/2 and 3T3-L1, respectively). SU6668 alone had no significant effect on expression of *Gli1*, *Ptch1* and *Gli2* genes. Expression of *Ulk3* was elevated approximately 1.5–2 times in the presence of 20 μM SU6668 (Fig. 2A d and Supplementary Fig. 2A d).

Shh pathway transcriptional targets *Gli1* and *Ptch1* became strongly induced in the Shh-stimulated cells: more than 100 and 30 times above the control, respectively. In order to synchronize data obtained from independent experiments, expression levels of *Gli1* and *Ptch1* in the cells treated with Shh and DMSO were set as 100%, and the data from samples treated with Shh and SU6668 were calculated relative to this (Fig. 2A a, b). SU6668 inhibited activation of *Gli1* and *Ptch1* expressions in a concentration dependent manner. In response to Shh, the levels of *Gli2* and *Ulk3* transcripts increased approximately 3 and 1.5 times, respectively (Fig. 2A c, d). In contrast to *Gli1* and *Ptch1*, expression levels of *Gli2* and *Ulk3* were not significantly down-regulated by SU6668.

Along with RNA analysis, Gli1 and Gli2 protein levels were analyzed by WB (Fig. 2B and Supplementary Fig. 2B). Shh stimulated an increase of Gli2 protein level that correlated with up-regulation of *Gli2* mRNA expression (Figs. 2B, upper panel, lanes 1 and 4 and A c). Nevertheless, the levels of *Gli2* protein and mRNA did not correlate in the SU6668-treated cells. The level of *Gli2* mRNA was similar in the cells treated with either 10 or 20 μM SU6668 alone or combined with Shh (Fig. 2A c). In contrast, cells treated with Shh and SU6668 expressed less Gli2 protein compared with the cells treated only with SU6668 (Fig. 2B, upper panel, lanes 3, 6 and 7). Generally, the levels of Gli2 protein, but not mRNA, correlated with the levels of Gli2 transcriptional target *Gli1* in the cells treated with SU6668 and Shh (Fig. 2A a). The specificity of Gli2 antibodies was tested using C3H10T1/2, MDA-MB-231 and peripheral blood mononuclear cells (PBMCs) of mouse and human origin. In contrast to other cells analyzed, PBMCs do not express *Gli2* mRNA, but express other components of the Shh signaling pathway (Supplementary Fig. 1A). Gli2FL migrating at approximately 165–170 kDa was detected using immuno-blotting in C3H10T1/2 and MDA-MB-231 cells but not in PBMCs (Supplementary Fig. 1B–C).

Although *Gli1* transcripts were detected in the control cells, Gli1 protein was undetectable in the absence of Shh (Fig. 2B, middle panel). Similarly to *Gli1* mRNA and Gli2 protein, Gli1 protein was up-regulated in response to Shh and down-regulated in the presence of SU6668.

It has been shown that stimulation of cells with Shh leads to rapid stabilization and nuclear translocation of Gli2FL [7]. Gli2FL protein was examined by WB in whole cell and nuclear extracts (WCE and NE, respectively) of C3H10T1/2 cells treated with 20 μM SU6668 and/or Shh for 2 h (Fig. 2C). The amount of Gli2FL increased in response to Shh in WCE and NE signifying physical stabilization and nuclear translocation of Gli2FL. SU6668 inhibited the positive effect of Shh on Gli2FL indicating that one of the possible mechanisms of SU6668 action may be prevention of stabilization and/or nuclear transport of Gli2FL. In order to confirm the visual observations, Gli2 and Lamin B and/or GAPDH immuno-blotting images obtained from 3 independent experiments were quantified. Gli2 protein levels were normalized using Lamin B or GAPDH protein levels and set as 1 in the control sample. Data from other samples were calculated relative to that (Fig. 2D). The level of Gli2 protein increased approximately 1.6 times in response to Shh in WCE and NE and remained similar to the control level in the presence of SU6668.

### 3.3 SU6668 acts *via* Ulk3

**Fig. 3:**
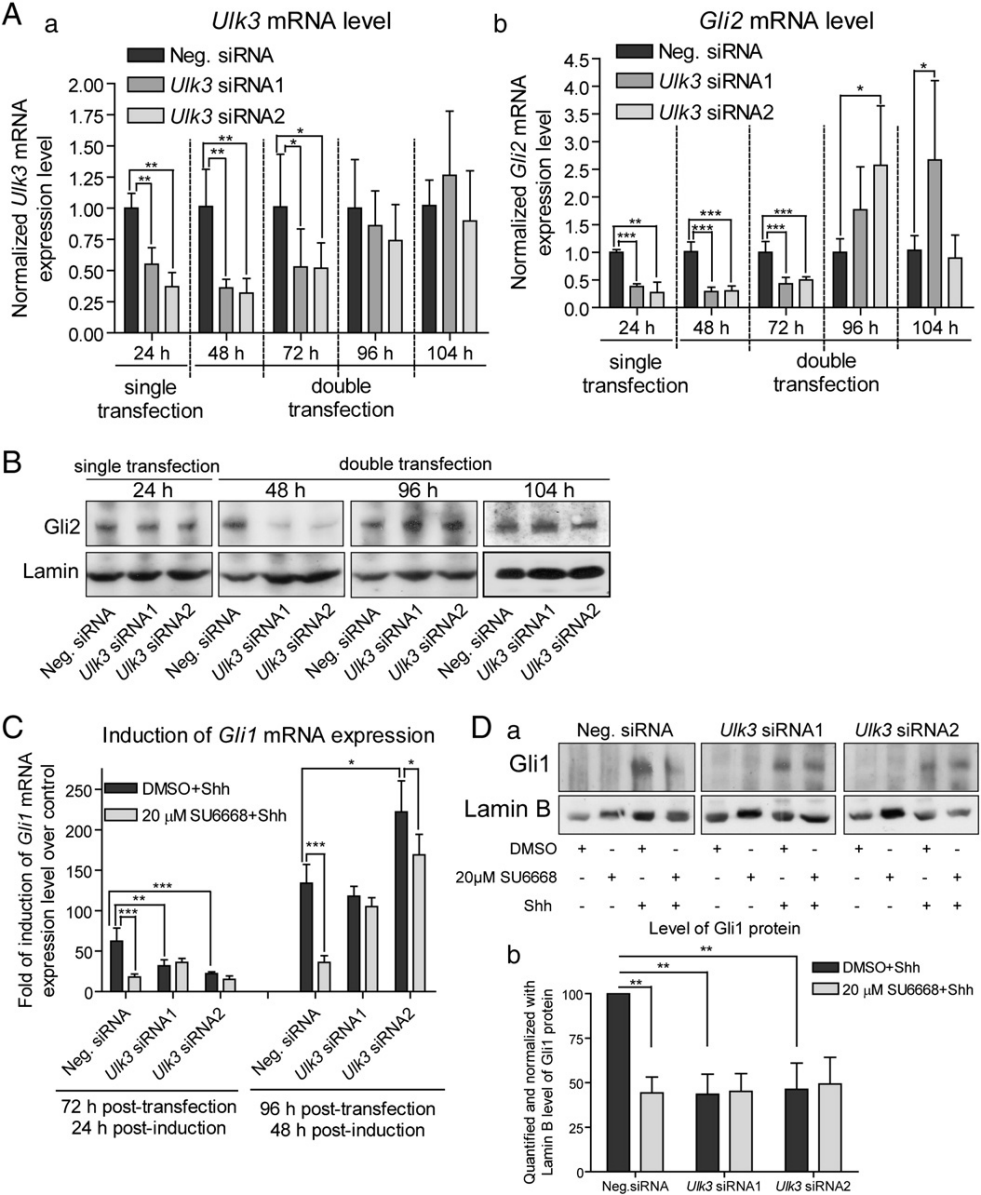
SU6668 inhibits Shh dependent up-regulation of Gli1 *via* Ulk3. (A, B, C, D) 3 T3-L1 cells were transfected with *Ulk3*-specific Silencer® Select siRNAs S89965 and S89966 using reverse followed by forward procedures resulted in single and double transfection, respectively. The cells were treated with DMSO, 20 μM SU6668 and Shh, if specified, and incubated during the indicated periods of time. (A) Levels of *Ulk3* (a) and *Gli2* (b) transcripts were measured by qPCR, normalized with *Hprt* mRNA expression level and set as 1 in the cells transfected with Negative siRNA. The data from samples transfected with *Ulk3*-specific siRNAs were calculated relative to the negative control. (B) The cells were subjected to WB analysis after the indicated periods of time of the siRNA delivery using Gli2 G-20 antibody. (C) The cells subjected to double transfection by siRNAs were treated with Shh, DMSO or SU6668 for 24 and 48 h. *Gli1* mRNA expression level was measured by qPCR, normalized with *Hprt* expression level and set as 1 in the respectively transfected control sample treated with DMSO. *Gli1* mRNA expression level in the Shh-treated samples were calculated relative to the control. The data are presented as fold of induction of *Gli1* mRNA level. Data of qPCR analyses are presented as an average mean ± S.D.; *—p < 0.05, **—p < 0.01, ***—p < 0.001, n = 3. (D, a–b) Lysates of 78 h post-transfectional and 30 h post-inductional cells were analyzed by WB. Gli1 and Lamin B images were quantified. The normalized Gli1 protein level in the control cells treated with DMSO and Shh was set as 100%. Data from other samples were calculated relative to that. Data are presented as an average mean ± S.D.; **—p < 0.01, n = 4.

**Supplementary Fig. 3:**
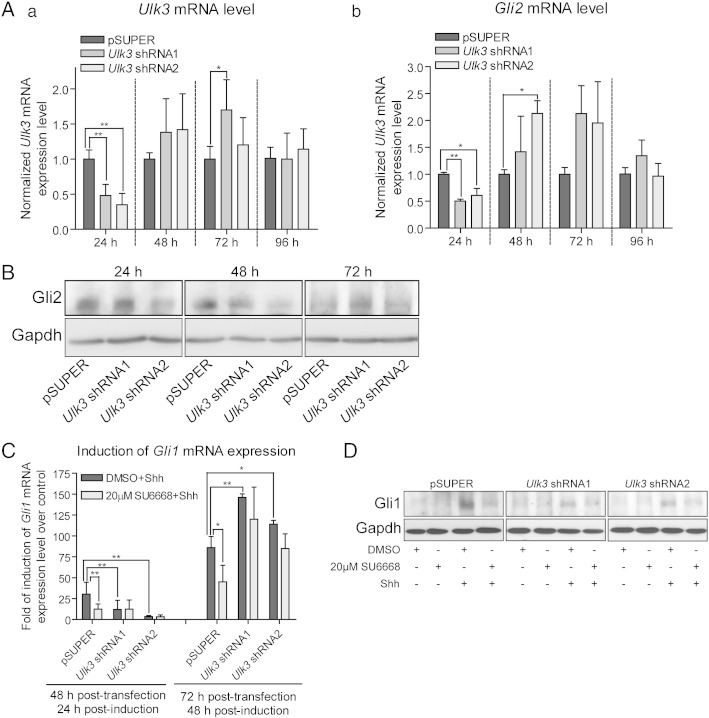
(A) C3H10T1/2 cells were transfected with empty vector pSUPER or *Ulk3* shRNAs and incubated during the indicated periods of time. Expression levels of *Ulk3* (a) and *Gli2* (b) were analyzed using qRT-PCR. The normalized with *Hprt* level of the respective gene expression at the indicated time point was set as 1 in the control cells transfected with pSUPER, and the data from the samples transfected with *Ulk3* shRNAs were calculated relative to the control. The data are presented as an average mean ± S.D. (B) Expression of Gli2 and GAPDH proteins was analyzed by Western-blotting at the indicated time points in the post-transfectional C3H10T1/2 cells electroporated with empty vector pSUPER or *Ulk3* shRNAs. *Gli2* was detected using AF3635 antibody. (C) C3H10T1/2 cells were electroporated with pSUPER or *Ulk3* shRNAs encoding constructs, incubated for 24 h and treated with DMSO or SU6668 in the presence or absence of Shh for additional 24 h or 48 h. *Gli1* mRNA expression level was measured by qRT-PCR after 24 h and 48 h of treatment initiation (after 48 h and 72 h of transfection, respectively). For each data set obtained from the cells transfected with the same construct, the level of *Gli1* mRNA expression was accepted as 1 in the control samples treated with DMSO. Data from other samples were calculated relative to their respectively transfected controls. The data are presented as an average mean of induction of *Gli1* mRNA expression over control ± S.D. (D) C3H10T1/2 cells were transfected with pSUPER or *Ulk3* shRNAs encoding constructs, incubated for 24 h and treated as indicated for additional 48 h. Levels of Gli1 and GAPDH proteins were examined by immuno-blotting (48 h post-treatment, 72 h post-transfection); *—p < 0.05, **—p < 0.01, n = 3.

It has been shown that SU6668 induces G2/M phase arrest of HeLa cells [23]. Block of cell cycle in G2/M phase and subsequent inhibition of primary cilia formation may negatively influence the Shh pathway execution. Therefore, the cell cycle profile was analyzed in C3H10T1/2 and 3T3-L1 cells treated with Shh and/or different concentrations of SU6668. No significant change in G2/M phase of cell cycle was detected (Supplementary Fig. 2D). It has been shown that several other kinases apart of Ulk3 participate in regulation of Gli proteins, and SU6668 is not specific inhibitor for Ulk3 [22], [23]. In order to examine, whether the effects of SU6668 on Gli proteins are mediated *via* Ulk3 kinase, we suppressed *Ulk3* mRNA expression in C3H10T1/2 and 3T3-L1 cells. For *Ulk3* RNAi, the previously described pSUPER constructs encoding *Ulk3* shRNAs [6] and *Ulk3*-specific synthetic siRNAs S89965 and S89966 (Ambion) were used. C3H10T1/2 cells were electroporated with the constructs encoding *Ulk3* shRNAs, and 3T3-L1 cells were transfected with *Ulk3* synthetic siRNAs using Lipofectamine. The obtained results are depicted in Fig. 3 and Supplementary Fig. 3 for 3T3-L1 and C3H10T1/2 cells, respectively.

*Ulk3* and *Gli2* mRNA expression levels were measured using qPCR and set as 1 in the cells transfected with Neg. siRNA (Fig. 3A a, b). *Ulk3* mRNA levels measured at 24 h post-transfection were reduced approximately 45% and 64% in the 3T3-L1 cells transfected with *Ulk3* siRNA1 and siRNA2, respectively. The levels of *Gli2* mRNA measured in the same cells were reduced approximately 62% and 73%. Cells transfected with *Ulk3* siRNAs twice expressed approximately 70% less *Ulk3* and *Gli2* transcripts. Initial low *Ulk3* and *Gli2* mRNA levels increased gradually during propagation of cells reaching and even exceeding the control levels. Similar tendency was observed in C3H10T1/2 cells; however, the increase of *Ulk3* and *Gli2* mRNA levels was more rapid (already within 48 h after transfection) probably due to the single electroporation procedure applied (Supplementary Fig. 3A a–b).

Gli2 protein level was assessed by WB. Correlation between the levels of *Gli2* protein and mRNA was observed with some temporal shift. Although 24 h post-transfectional cells contained similar amounts of Gli2 protein, cells transfected twice with *Ulk3* siRNAs demonstrated reduced levels of Gli2 protein (Fig. 3B). Gli2 protein level gradually increased in the cells transfected with *Ulk3* siRNAs during propagation exceeding that of control 96 h after initial transfection which correlated with up-regulation of *Gli2* mRNA level.

Afterwards, 48 h post-transfectional cells expressing the lowest levels of *Gli2* and *Ulk3* were treated with Shh, 20 μM SU6668 or DMSO for 24 and 48 h. *Gli1* mRNA levels were measured by qPCR. For the each particular set of data, *Gli1* expression level was considered as 1 in control cells treated with DMSO and the data from other respectively transfected but differently treated samples were calculated as fold of *Gli1* mRNA induction over control. SU6668 alone had no significant effect on *Gli1* mRNA expression (data not shown). In response to Shh, *Gli1* mRNA level initially increased approximately 75 times in the control cells and 32 and 22 times in the cells transfected with *Ulk3* siRNAs (Fig. 3C). However, compared to the control cells, *Gli1* mRNA level became similar or even higher in the cells transfected with *Ulk3* siRNAs at the next time point (96 h post-transfection, 48 h post-induction with Shh). As the above-described analyses revealed, *Gli2* mRNA and protein levels had been also increased at this time point in these cells (Fig. 3A b and B) that may be associated with increased transcriptional activity of Gli2. Up-regulation of Gli2 may explain higher *Gli1* mRNA expression levels in the cells transfected with *Ulk3* siRNAs and induced by Shh for 48 h. SU6668, added together with Shh, was active in the control cells but could not effectively inhibit *Gli1* mRNA expression in the cells with reduced levels of *Ulk3* and *Gli2* (Fig. 3C). However, low negative effects of SU6668 on Shh dependent activation of *Gli1* in the case of restored levels of Gli2 observed 96 h after *Ulk3* RNAi may be caused by possible instability of SU6668 in cell culture conditions during 48 h of incubation and consequently, insufficient concentration of active SU6668 per Gli2/Ulk3 complex in the cells expressing increased levels of Gli2 protein.

In order to confirm the observations of the impact on *Gli1* mRNA level, Gli1 protein was analyzed by WB in the cells transfected twice with *Ulk3* or Neg. siRNAs and treated with Shh and/or 20 μM SU6668 for 32 h. Gli1 and Lamin B immuno-blotting images obtained from four independent experiments were quantified. Gli1 protein level was normalized with Lamin B and set as 100% in control sample. Data from other samples were calculated relative to this. Increase of Gli1 protein was stronger in the control cells, and SU6668 was not effective in the cells transfected with *Ulk3* siRNAs (Fig. 3D). Similar results were obtained analyzing levels of *Gli1* mRNA and protein in another experimental model (Supplementary Fig. 3C–D).

### 3.4 Regulation of *GLI1/2* by TGF-β and SU6668 in human immortalized breast cancer cells

**Fig. 4:**
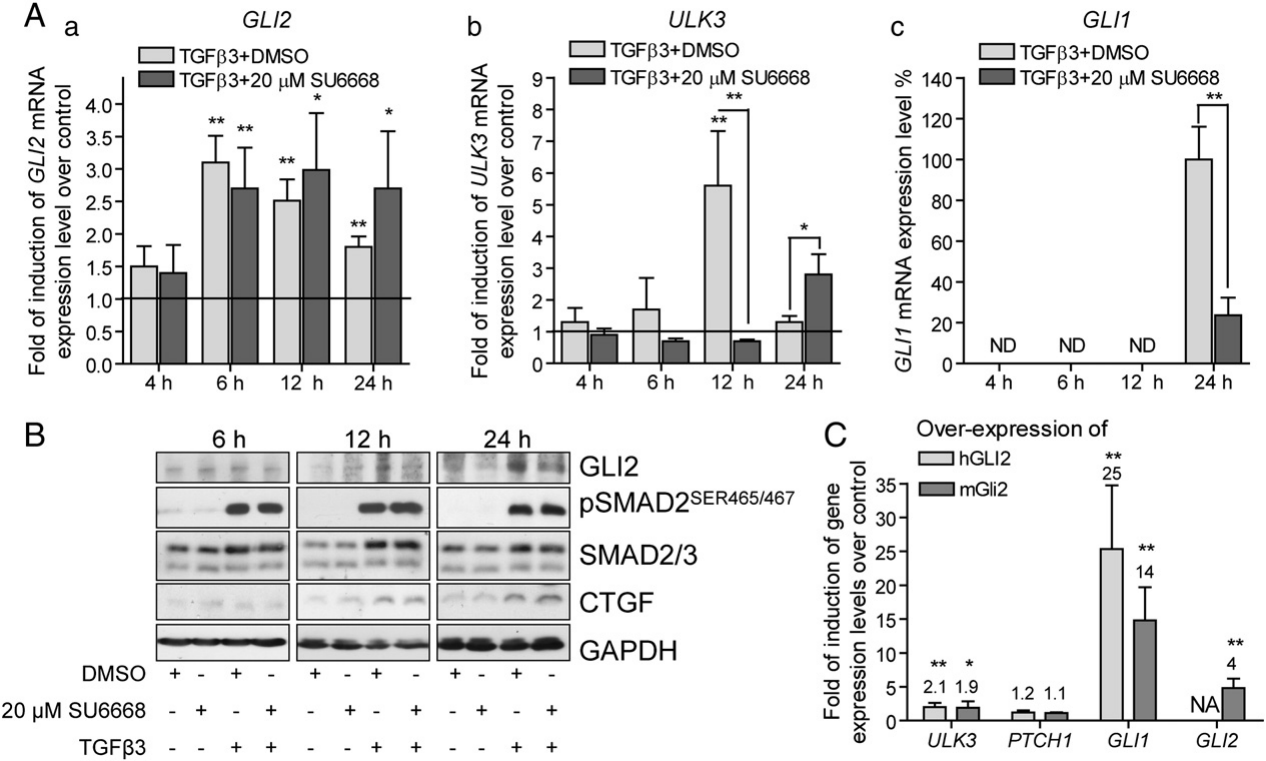
TGF-β induced positive regulation of GLI2 protein is inhibited by SU6668 in MDA-MB-231 cells. (A, B) MDA-MB-231 cells were treated with DMSO or 20 μM SU6668 in the presence of TGF-β3 during the indicated periods of time. (A) *GLI2* (a), *ULK3* (b) and *GLI1* (c) mRNA levels were analyzed by qPCR, normalized with *GAPDH* expression level and set as 1 in the control cells treated with DMSO (indicated by baseline on the panels a and b). The level of *GLI1* mRNA expression in the cells treated with TGF-β was set as 100%. The data are presented as an average fold of induction of gene expression level over a control ± S.D. (B) Whole cell lysates were subjected to WB analysis. GLI2 protein was detected using AF3635 antibody. (C) MDA-MB-231 cells were transfected either with plasmids encoding mouse mGli2 or human hGLI2. The levels of the indicated gene expression were measured by qPCR after 18 h. The data are presented as an average fold of induction ± S.D. of the particular gene expression level over a control level measured in the cells transfected with an empty vector; (A, C) *—p < 0.05, **—p < 0.01, ***—p < 0.001, n = 3, ND — not detected.

**Supplementary Fig. 4:**
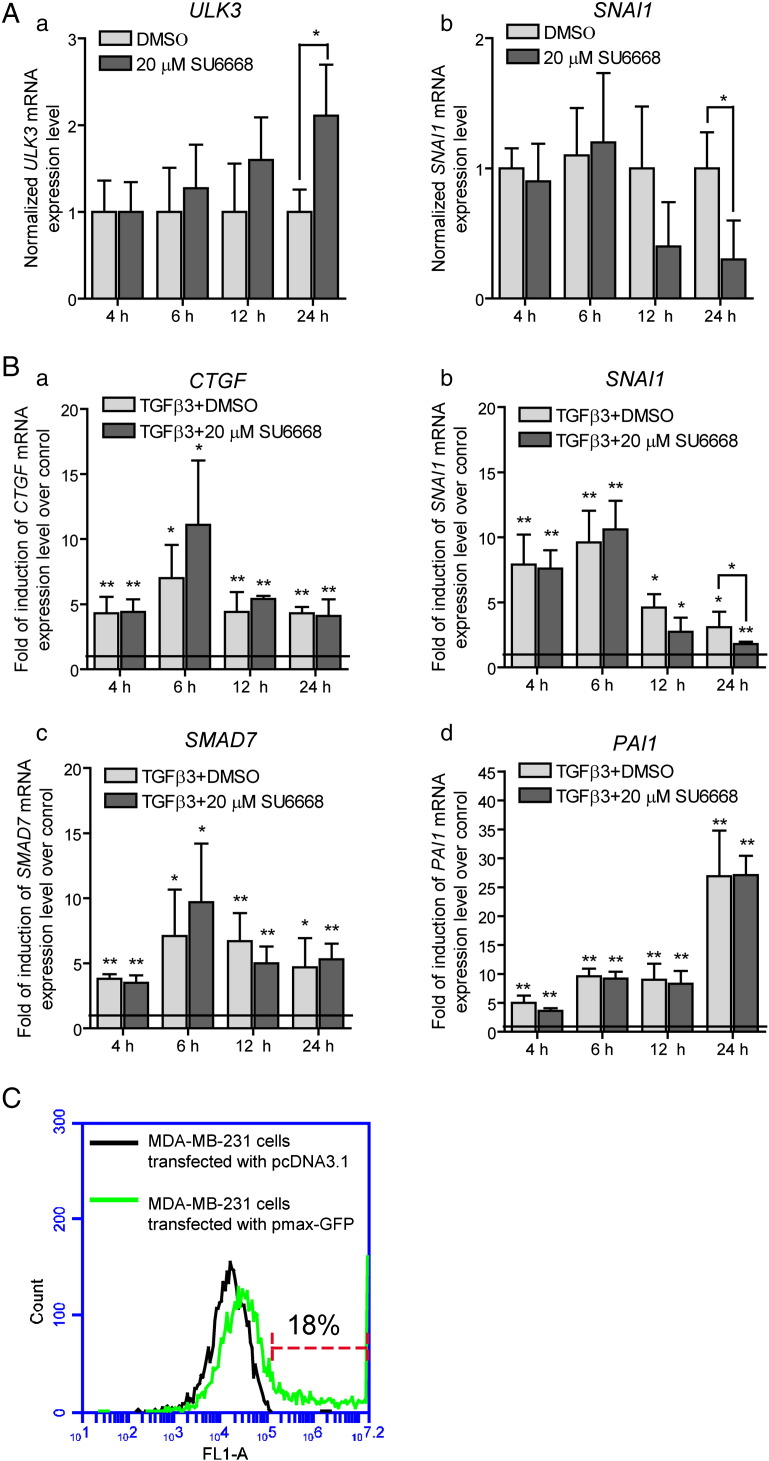
(A, B) MDA-MB-231 cells were treated with 10 ng/ml of TGF-β3 in the presence of DMSO or 20 μM SU6668 during the indicated periods of time. Expression levels of *ULK3*, *SNAI1*, *CSMAD7* and *PAI* were analyzed by qPCR, normalized with *GAPDH* mRNA levels and set as 1 in the control cells treated with DMSO at the respective time point (indicated as a baseline on panel B). The data from other samples were calculated relative to control. The data are presented as an average mean ± S.D.; *—p < 0.05, **—p < 0.01. (C) MDA-MB-231 cells were transfected with pcDNA3.1 or pmax-GFP vectors using Lipofectamine 2000. A relative number of GFP-positive cells were measured using Accuri C6 flow cytometer.

Apart from Shh, TGF-β regulates expression and transcriptional activity of GLI1/2 in a SMAD3, TCF4 and β-catenin dependent manner [18], [19]. In order to explore the effects of SU6668 on regulation of GLI2 in the context of TGF-β pathway, immortalized breast cancer cells MDA-MB-231 were used. These cells have been previously reported to express *GLI1*, *GLI2* and *PTCH1* and respond to TGF-β1 treatment with increased levels of *GLI2* and *GLI1* mRNAs [18], [43]. MDA-MB-231 cells were treated with TGF-β3, 20 μM SU6668 or DMSO for 4, 8, 12 and 24 h. The expression levels of “Shh mediators” *GLI2*, *GLI1* and *ULK3* and “classical” TGF-β target genes *SMAD7*, *PAI1*, *CTGF* and *SNAI1* were measured by qPCR and normalized to 1 in the cells treated with DMSO (Fig. 4A and Supplementary Fig. 4).

SU6668 had no statistically significant effect on expression of most genes analyzed except *ULK3* and *SNAI1* (data not shown and Supplementary Fig. 4A a, b). *ULK3* expression gradually increased during exposure of cells to SU6668 reaching almost 2-fold induction within 24 h. Expression of *SNAI1* was down-regulated by SU6668 approximately 65%.

TGF-β stimulated expression of all analyzed genes, however, the dynamic of up-regulation and effects of SU6668 varied for different genes (Fig. 4A and Supplementary Fig. 4A). Significant up-regulation of *SMAD7*, *PAI1*, *CTGF* and *SNAI1* was observed already within 4 h of TGF-β treatment. Expression of *GLI2* increased approximately 3 times during 6 h of TGF-β treatment and afterwards decreased gradually but remained elevated over control.

SU6668 did not significantly inhibit TGF-β induced up-regulation of *GLI2*, *SMAD7*, *PAI1* and *CTGF*. It had statistically insignificant positive effect on *GLI2* mRNA expression during prolonged propagation of cells in the presence of TGF-β (Fig. 4A a). Expression of *ULK3* was induced 12 h after activation of TGF-β signaling (Fig. 4A b). Increase of *ULK3* transcription was accompanied by augmentation of GLI2 protein level (Fig. 4A b and B, respectively). In contrast to “classic” TGF-β target genes, up-regulation of *ULK3* expression was inhibited by SU6668 that correlated with depleted level of GLI2 protein in the respectively treated cells. However, *ULK3* mRNA level increased in response to prolonged treatment with TGF-β and SU6668 similarly as it was observed in the case of SU6668 alone (Fig. 4A b and Supplementary Fig. 4A a). Also, TGF-β dependent induction of *SNAI1* expression was down-regulated by SU6668 suggesting a positive correlation between GLI2 transcriptional activity and expression of *SNAI1*.

Contrary to the previously published reports, *GLI1* expression was under the detection limit in native MDA-MB-231 cells. In order to compare *GLI1* mRNA levels induced by TGF-β in the presence or absence of SU6668, expression level of *GLI1* was set as 100% in the samples treated with TGF-β and the data from other samples were calculated relative to that. In contrast to *GLI2*, *GLI1* expression was significantly down-regulated by SU6668 24 h after treatment with TGF-β, as it was observed for *ULK3* at 12 h (Fig. 4A c).

WB analysis of cells treated with TGF-β combined with either DMSO or SU6668 showed similar levels of primary mediators of TGF-β signal — SMAD2/3 and activated form of SMAD2 phosphorylated at serine residues 465/467, as well as CTGF (Fig. 4B). However, GLI2 protein was not up-regulated in the presence of SU6668.

Increase of *ULK3* mRNA expression correlated with up-regulation of GLI2 protein level at the respective time point. These data suggest a possible involvement of GLI2 in regulation of *ULK3* gene expression. To test this hypothesis, mouse and human Gli2 were over-expressed in MDA-MB-231 cells for 16 h. The levels of *ULK3*, *PTCH1*, *GLI1* and *GLI2* mRNAs were measured by qPCR (Fig. 4C). GLI2 of human origin demonstrated slightly higher transcriptional activity than mouse Gli2 in MDA-MB-231 cells. Gli2 could up-regulate *GLI2* expression, either directly or *via* induction of GLI1. Over-expressed Gli2 proteins could not induce *PTCH1* expression in MDA-MB-231 cells, whereas *ULK3* expression was induced approximately 2 times suggesting that GLI2 may play a co-activator role in regulation of *ULK3* gene expression. Lower increase of ULK3 expression in response to over-expressed Gli2 proteins may be caused by poor transfection efficiency of MDA-MB-231 cells (approximately 18%) (Supplementary Fig. 4C).

### 3.5 ULK3 controls GLI1/2 proteins in human ASCs

**Fig. 5:**
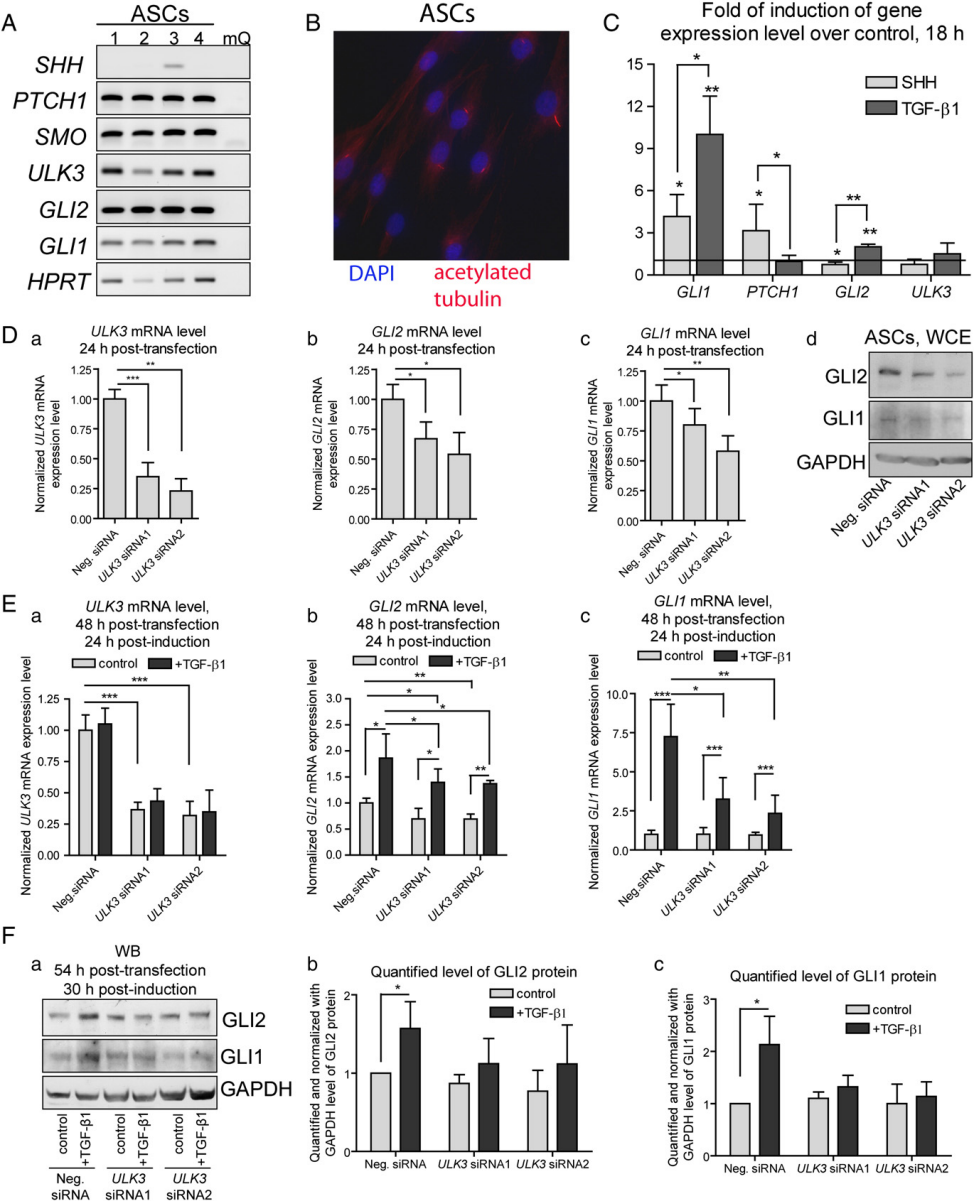
ULK3 participates in regulation of GLI1/2 transcriptional activators in ASCs. (A) Expression of genes encoding mediators of the Shh signaling pathway was analyzed using RT-PCR in ASCs isolated from four donors. (B) Primary cilia (red) and nuclei (blue) were stained using antibody against acetylated tubulin and DAPI, respectively. (C) Levels of *GLI1* (a), *PTCH1* (b), *GLI2* (c) and *ULK3* (d) mRNAs were measured by qPCR in ASCs stimulated with Shh or TGF-β1. (D) ASCs were transfected with Neg. or *ULK3*-specific siRNAs. The levels of *ULK3* (a), *GLI2* (b) and *GLI1* (c) transcripts were measured by qPCR; (d) GLI1 and GLI2 proteins were analyzed by immuno-blotting. (E) ASCs were transfected with Neg. or *ULK3*-specific siRNAs and 24 h later treated with TGF-β1 for 24 h. The levels of *ULK3* (a), *GLI2* (b) and *GLI1* (c) transcripts were measured by qPCR. (F) (a) GLI2 and GLI1 proteins were analyzed by WB in cells transfected with Neg. or *ULK3*-specific siRNAs and induced with TGF-β1 for 30 h. GLI2, GLI1 and GAPDH images were quantified. Normalized GLI2 (b) and GLI1 (c) protein levels were set as 1 in cells transfected with Neg. siRNA. Data of qPCR and quantified WBs are presented as an average mean ± S.D.; *—p < 0.05, **—p < 0.01, ***—p < 0.001, n = 3.

ASCs represent heterogeneous populations of cells able to respond to variable stimuli by expressing different lineage-specific markers and differentiate, at least under *in vitro* conditions, towards adipocytes, chondrocytes and osteoblasts [24]. Since human ASCs have not been systematically described in respect of the canonical Shh pathway, expression of the mediators of Shh signaling (*SHH*, *PTCH1*, *SMO*, *ULK3*, *GLI2* and *GLI1*) was examined by RT-PCR in ASCs isolated from four donors. All genes except *SHH* were expressed in ASCs (Fig. 5A). Very low level of *SHH* expression was detected only in one sample suggesting that generally ASCs are not subjected to autocrine regulation by Shh. Also, ASCs were stained using an antibody against acetylated tubulin to visualize primary cilia required for the canonical Shh signal transduction. Approximately 50% of ASCs displayed primary cilia (Fig. 5B).

GLI proteins can be activated *via* Shh and TGF-β signaling axes. In order to examine canonical and non-canonical activation of GLI1/2 proteins in ASCs, the cells were treated with Shh or TGF-β1 for 18 h and the levels of expression of *GLI1*, *PTCH1*, *GLI2* and *ULK3* were examined by qPCR. The cells responded to Shh treatment by increased level of *GLI1* and *PTCH1* mRNA, whereas *GLI2* mRNA level was elevated in response to TGF-β (Fig. 5C). In all cells analyzed, TGF-β activated *GLI1* transcription approximately 2.2 times stronger than Shh did probably due to associated activation of *GLI2*. Up-regulation of *GLI1* and *PTCH1* expression in response to Shh, presence of primary cilia and expression of the key mediators of the Shh signaling together strongly indicate a subpopulation of ASCs possessing the functional Shh pathway.

Next, ULK3 was assessed as a regulator of GLI1/2 in ASCs. *ULK3* mRNA expression was inhibited approximately 65 and 77% using two synthetic siRNAs (Fig. 5D a). Concurrently, *GLI2* and *GLI1* mRNA and protein levels were reduced in these cells (Fig. 5D b–d). Of note, in contrast to the mouse cells used, the level of GLI1 protein was high enough to be detected by WB in native ASCs (Fig. 5D d). Afterwards, 24 h post-transfectional ASCs were treated with TGF-β1 for additional 24 h and expression of *ULK3*, *GLI2* and *GLI1* were analyzed by qPCR. Gene expression levels in control cells transfected with Neg. siRNA were set as 1, and data from other samples were calculated relative to this (Fig. 5E a–c). The levels of *ULK3* transcripts remained reduced 48 h post-transfection in cells treated with *ULK3* siRNAs. *GLI2* expression was induced in response to TGF-β treatment approximately 1.7 times in all cells analyzed. However, the absolute levels of *GLI2* transcripts remained lower in the TGF-β1 treated cells transfected with *ULK3*-specific siRNAs and expressed lower levels of *GLI2* mRNA and protein at treatment initiation. Substantial increase of GLI2 protein level in response to TGF-β1 was observed only in ASCs transfected with Neg. siRNA (Fig. 5F a, upper panel and b). *GLI1* transcription was induced by TGF-β1 approximately 7.3, 2.6 and 2.4 times in ASCs transfected with Neg. siRNA, *ULK3* siRNA1 and siRNA2, respectively (Fig. 5E c). Similarly to GLI2, significant increase of GLI1 protein in response to TGF-β treatment was observed only in the cell transfected with Neg. siRNA (Fig. 5F). Taken together, these data indicate that ULK3 does not participate in the TGF-β signaling cascade but is required for stability of GLI2 and GLI1 proteins.

### 3.6 SU6668 inhibits osteo- and chondrogenic differentiation of ASCs

**Fig. 6:**
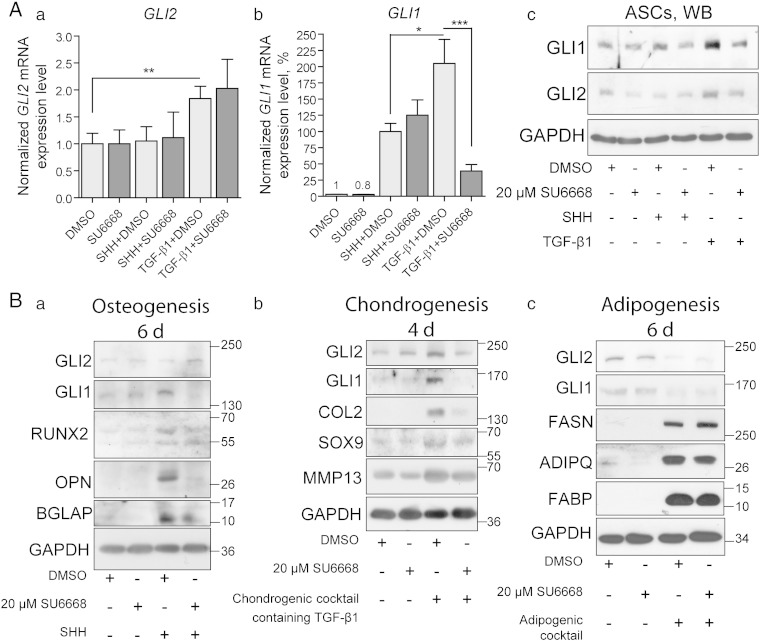
SU6668 prevents *de novo* generation of GLI1 and GLI2 proteins in ASCs. (A) ASCs were treated with Shh, TGF-β1 and 20 μM SU6668 or DMSO. (a) Levels of *GLI2* and *GLI1* transcripts were measured using qPCR. Data are presented as an average mean ± S.D.; *—p < 0.05, **—p < 0.01, ***—p < 0.001, n = 4. (b) WCEs were subjected to immuno-blotting analysis. (B) ASCs were treated with Shh (a), chondrogenic cocktail containing TGF-β1 (b) or adipogenic cocktail containing insulin, DEX, IBMX and indomethacin (c) in the presence of DMSO or 20 μM SU6668. GLI1/2 and lineage specific proteins were analyzed by WB.

In order to explore the effect of SU6668 on GLI1/2 proteins in primary human cells, ASCs were treated with Shh or TGF-β1 in the presence of 20 μM SU6668 or DMSO. *GLI1* and *GLI2* expression levels were assessed by qPCR and the respective protein levels by immuno-blotting (Fig. 6A a–c). In order to synchronize the data obtained from different experiments, *GLI1* expression level in control ASCs treated with Shh was set as 100% and data from other samples were recalculated relative to this. TGF-β stimulated *GLI2* mRNA expression was not suppressed by SU6668, as it was observed in MDA-MB-231 cells (Figs. 6A a and 4A a, respectively). Also, SU6668 had no negative effect on Shh-induced *GLI1* mRNA expression but inhibited expression of *GLI1* up-regulated in response to TGF-β signaling (Fig. 6A b). However, immuno-blotting analysis showed that induction of GLI1 and GLI2 proteins in response to Shh or TGF-β was restrained by SU6668 (Fig. 6A c). The behavior of *GLI1* in ASCs challenged with Shh and SU6668 resembled that of *Gli2* in mouse C3H10T1/2 and 3T3-L1 cells: *GLI1* mRNA and protein levels did not correlate in ASCs treated with Shh and SU6668 similarly to non-correlated *Gli2* mRNA and protein levels in the respectively treated mouse progenitor cells (Figs. 6A b and c 1st panel, 2A c, B 1st panel, Supplementary Fig. 2A c and Supplementary Fig. 2B 1st panel). Similar phenomenon was observed in the case of *GLI2* and TGF-β: *GLI2* transcription was not inhibited by SU6668, but up-regulation of GLI2 protein and as a result its transcriptional activity was restrained. These data reveal that effects of SU6668 resemble effects of *ULK3* RNAi in the case of GLI1/2 activation: neither SU6668 nor *ULK3* knockdown interrupts Shh or TGF-β signaling cascades but both reduce stability of the *de novo* generated GLI1 and/or GLI2 proteins (Fig. 5 E–F and 6A). The difference between *ULK3* RNAi and SU6668 treatments becomes apparent in the case of regulation of basal GLI proteins: SU6668 has no significant effect on their expression, but *ULK3* knockdown leads to decrease of basal GLI1/2 levels (Fig. 5D and 6A c). This difference may be explained by possible dual role of ULK3: SU6668 inhibits only catalytic activity of ULK3 kinase, whereas both regulatory and catalytic activities are disrupted by *ULK3* RNAi.

**Supplementary Fig. 5:**
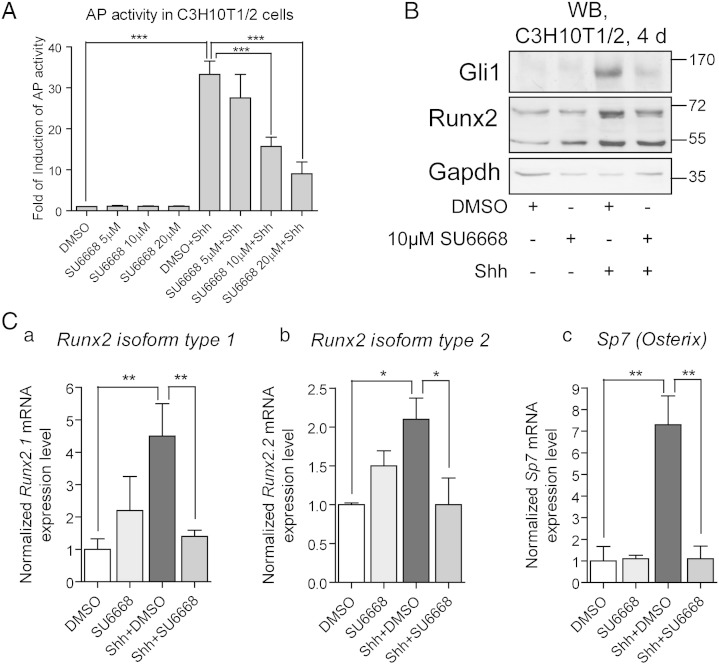
SU6668 prevents Shh-induced osteogenic differentiation of C3H10T1/2 cells. (A) C3H10T1/2 cells were treated as indicated for 3 d. Alkaline phosphatase (AP) activity was measured and normalized with the amount of total protein. The normalized level of AP activity in the control sample treated with DMSO was set as 1. The normalized data are expressed as an average mean ± S.D. ***—p < 0.001, n = 4. (B, C) C3H10T1/2 cells were treated for 4 d. Whole cell extracts were subjected to WB analysis. The levels of the respective gene mRNA expression were measured by qPCR, normalized with *Hprt* mRNA expression levels and set as 1 in the samples treated with DMSO. The data are presented as an average mean ± S.D. *—p < 0.05, **—p < 0.01, ***—p < 0.001, n = 3.

To study the effects of SU6668 on tri-lineage differentiation of human ASCs, the respective treatments were conducted for 4 or 6 d, and WCEs were analyzed using immuno-blotting. Shh triggered expression of all osteogenic differentiation markers tested (both RUNX2 isoforms, OPN and BGLAP) and GLI1 (Fig. 6B a). Chondrocytes specific proteins COL2, SOX9, MMP13 as well as GLI1 and GLI2 were induced in response to prolonged exposure to TGF-β1 containing media (Fig. 6B b). SU6668 inhibited expression of the chondro- and osteo-lineage specific markers in ASCs, as it was observed in C3H10T1/2 cells (Fig. 6B and Supplementary Fig. 5, respectively). The inhibiting effect of SU6668 was noticeable in the case of the higher molecular weight RUNX2 isoform that was induced to higher extent by Shh (Fig. 6B a 3rd panel and Supplementary Fig. 5B–C). In contrast to osteo- and chondrogenic differentiation programs, adipogenic differentiation of ASCs was accompanied by down-regulation of GLI1/2 protein and was not inhibited by SU6668 (Fig. 6B c).

## 4 Discussion

Regulation of Gli1/2 transcription factors is one of the cross-talking points of Shh and TGF-β signaling pathways. However, mechanistic differences specific for these intracellular signaling cascades are obvious: TGF-β/SMAD axis controls *GLI2* transcription, whereas post-translational regulation of Gli2 protein activity is the central event in the Shh pathway. In both cases, Gli2 is known to up-regulate *Gli1* transcription. In this study we have identified SU6668 as a small molecular weight modulator of Gli protein activity and shown that there are common elements in regulation of Gli1/2 transcriptional activators by TGF-β and Shh.

### 4.1 SU6668 as an inhibitor of Ulk3 kinase activity and *de novo* generated Gli proteins

The existing inhibitors of the Shh pathway mostly target SMO oncoprotein, the trans-membrane signaling effectors of Shh. However, in many cases the somatic gain-of-function mutations in *SMO* occur in human cancers, making SMO inhibitors useless for therapeutic purposes [21]. Also, the fact that GLI transcription factors may be functioning independently of the canonical Shh pathway requires development of novel inhibitors targeting activity of GLI proteins [44], [45], [46]. This kind of inhibitors might be useful in treatment of specific GLI-dependent types of cancer. ULK3 has been suggested as a regulator of GLI1/2 transcriptional activators able to interact with the ATP-competitive tyrosine kinase inhibitor SU6668 [23], [40].

Here, SU6668 is demonstrated as an inhibitor of ULK3 kinase activity *in vitro* with K_i_ of 0.2 μM. Biochemical K_i_ of SU6668 towards receptor tyrosine kinases ranged from 0.008 μM for PDGFR to 2.1 μM for VEGFR2, and IC_50_ values for a number of other kinases were at least 10 μM [22]. The observed K_i_ value indicates that SU6668 is effective inhibitor also for ULK3.

Our results demonstrate that Gli1/2 transcriptional activators may be suppressed by SU6668 in different types of cells and independently of the signaling cascade involved in their activation. First, using two mouse cell lines and *Ulk3* RNAi approach we have demonstrated that SU6668 inhibits the Shh pathway execution *via* Ulk3, as assessed by analyses of *Gli1* and *Ptch1* expression induced in a Gli2 dependent manner. Second, elevated levels of GLI1/2 proteins are down-regulated using SU6668 in ASCs. Third, SU6668 inhibits TGF-β mediated activation of GLI2 protein and subsequent *GLI1* and *ULK3* transcription in MDA-MB-231 cells. These findings are very important in the light of the established oncogenic properties of GLI1/2.

Interestingly, SU6668 does not inhibit TGF-β mediated transcriptional activation of *GLI2* in ASCs or MDA-MB-231 cells as well as Shh induced expression of *GLI1* mRNA in ASCs. A similar phenomenon was observed in Shh dependent up-regulation of *Gli2* expression in the presence of SU6668 in mouse cells. However, Gli1/2 protein levels were substantially down-regulated in all above-mentioned cases. The observed negative effect of SU6668 might be explained by inhibition of post-transcriptional mechanisms regulating translation or stability of *de novo* generated Gli1/2 proteins in the context of active Shh or TGF-β signaling pathways. The mechanism of SU6668 action on activated GLI1/2 is similar to effects of *ULK3* RNAi: activation of *GLI2* transcription was not interfered, but the levels of GLI2 protein were not elevated in response to TGF-β. These results let to assume that SU6668 acts *via* ULK3. Also, our *Ulk3* RNAi experiments revealed that SU6668 inhibits Gli proteins *via* Ulk3. Taken together these data suggest that Ulk3 is required not only for activation but also for stabilization of endogenous Gli1/2 proteins. This hypothesis is supported by data showing that Ulk3 is involved in maintenance of basal *Gli2* expression. In view of the fact that SU6668 is not a very specific inhibitor of Ulk3, it cannot be formally ruled out that other kinase(s) regulating Gli1/2 may be also inhibited.

It is noteworthy that *Ulk3* mRNA level increased during prolonged culturing of cells in the presence of SU6668. Because of lack of an Ulk3-specific antibody, we failed to examine a correlation between *Ulk3* protein and mRNA levels and may only speculate that this increase could be caused by indirect compensatory transcriptional up-regulation of *Ulk3*, since our previous work has suggested that Ulk3 kinase is required for maintenance of cellular homeostasis [6].

### 4.2 Generation of Gli2ACT

Studies on the dynamics of the Shh pathway activation have suggested a mechanism of Gli2/3ACT formation: in response to ligand binding, Gli2/3FL are stabilized and transformed to Gli2/3ACT probably *via* yet uncharacterized phosphorylation event [7], [12], [47]. Our analysis of Gli2 in whole cell and nuclear extracts also shows that Shh induces rapid stabilization of Gli2FL and increase of nuclear Gli2FL, where it probably acts as a transcriptional activator. It is plausible that the observed nuclear translocation of Gli2FL is a consequence of specific phosphorylation resulted in generation of Gli2ACT. Several serine/threonine kinases, such as PKA, GSK3, CK1 and DYRK2, can directly phosphorylate Gli2, but the functional consequence of these phosphorylations is connected to processing and degradation of Gli2 [48], [49]. Phosphorylation of Gli2 by Ulk3 leads to increase of its transcriptional activity [40]. Our data show that SU6668 abolishes the Shh-induced nuclear translocation of Gli2FL and suggest that the nuclear form of Gli2FL may be the one phosphorylated by Ulk3 and this kind of phosphorylation is required for Gli2ACT generation.

### 4.3 Interrelationship between Gli2 and Ulk3 activities

The present work has revealed that Ulk3 and Gli2 are interconnected and probably subjected to cross-regulation on transcriptional and/or post-translational levels. *Ulk3* RNAi resulted not only in reduction of *Ulk3* transcripts but also in loss of Gli2 in mouse cells and human ASCs, whereas TGF-β-induced endogenous or over-expressed GLI2 proteins were associated with induction of *ULK3* expression in MDA-MB-231 cells. These results indicate a possible positive feedback loop between Gli2 and Ulk3: Ulk3 mediated phosphorylation is required for stabilization and activation of Gli2, which in turn participates in positive regulation of *Ulk3* transcription. Bioinformatic analysis of 600 bp of the *ULK3* 5′UTR revealed at least 3 non-consensus Gli binding sites: GTCCTCCCA(− 591 bp), GGCCTCCCA(− 471 bp) and AGCCACCCA(− 78 bp) (non-consensus nucleotides are underlined), which may be functional, since the sites with single substitutions have activities similar to the consensus site in luciferase reporter assay [50]. Nevertheless, the issue whether the Gli2 protein binds directly to *Ulk3* promoter or works in an indirect manner remains to be investigated.

As it has been shown previously, inhibition of *Ulk3* expression using plasmids expressing *Ulk3*-specific shRNA led to stronger increase of *Gli1* mRNA expression in response to Shh than it was achieved in the control cells after 48 h of treatment [6]. The same phenomenon was observed in the present study in two experimental models and may be explained by up-regulation of *Ulk3* and *Gli2* to the levels higher than in the control cells at late stages of RNAi experiments. However, the present more detailed analysis has revealed that induction of *Gli1* by Shh is attenuated in the cells with reduced expression levels of *Ulk3* and *Gli2* at initial stages of treatment. These results confirm a previously suggested, but not experimentally tested, model assuming a positive role of Ulk3 in regulation of endogenous Gli proteins.

### 4.4 Shh triggers the osteogenic differentiation program in ASCs

ASCs represent a promising tool for cell therapy and regenerative medicine, areas strictly depending on detailed understanding of the molecular regulatory mechanisms controlling behavior and fate of transplanted cells.

The present study shows that human ASCs possess functional Shh signaling axis. ASCs respond to Shh by increasing the levels of *GLI1* and *PTCH1* expressions, although the extent of their activation was at least 10 times lower compared with the mouse cell lines studied. This may be explained by relatively small and variable fractions of cells positive for Shh co-receptors PTCH/SMO within the heterogeneous ASCs isolated from different donors. An interesting point to make from these experiments is that *GLI1* is found to be expressed at levels detectable by immuno-blotting in all ASCs analyzed. These data are in contrast to the mouse cells studied, where Gli1 protein cannot be detected without challenging the cells with Shh. The level of GLI1 protein was under the detection limit also in MDA-MB-231 cells even in the case of TGF-β treatment triggering up-regulation of *GLI1* mRNA expression. Contrary to mouse cells, Shh has no significant positive effect on GLI2 expression or stability in ASCs that may be effectively induced by TGF-β. These results raise the possibility that the Shh signaling cascade may utilize GLI1 instead of GLI2 as a primary transcriptional activator because of high basal GLI1 expression in ASCs.

The Hh/Gli axis is known to control commitment of multipotent cells towards chondrocyte and osteocyte lineages in mice [28], [51]. This study shows that prolonged exposure to Shh triggers expression of osteocytic lineage specific markers in ASCs. These observations suggest Shh co-receptors PTCH/SMO as possible surface markers for isolation of a subpopulation of ASCs capable for osteogenic differentiation, which potentially could be very useful for the branch of regenerative medicine involved in treatment of disorders caused by loss of bone tissue.

Treatment with SU6668 attenuates both TGF-β induced chondrogenic and Shh mediated osteogenic differentiation programs. These effects are consistent with the putative roles of GLI proteins in these differentiation paradigms. In contrast, differentiation of ASCs towards adipocytes was effective in the presence of SU6668. Although we are aware of the fact that SU6668 is not a very specific inhibitor of Ulk3, our results open up possibilities to design novel and more specific inhibitors of Ulk3 kinase and Gli proteins using SU6668 as a starting compound.

The following are the supplementary data related to this article

Supplementary Table 1 Donors of the cells used in the present study. ASCs — adipose tissue derived stromal cells; PBMCs — peripheral blood mononuclear cells.

Supplementary Table 2 List of antibodies.

Supplementary Table 3 List of primers.
