# Supplementary material for: Protein kinase inhibitor SU6668 attenuates positive regulation of Gli proteins in cancer and multipotent progenitor cells
Source: Biochim Biophys Acta. 2014 Apr;1843(4):703–14. doi: 10.1016/j.bbamcr.2014.01.003 (PMC3946003; doi:10.1016/j.bbamcr.2014.01.003)
Supplement: Supplementary Table 1 — Donors of the cells used in the present study. ASCs — adipose tissue derived stromal cells; PBMCs — peripheral blood mononuclear cells. [file mmc1.pdf]

Supplementary Table 1 Donors of the cells used in the present study
ASCs – adipose tissue derived stromal cells; PBMCs – peripheral blood mononuclear cells

Population	*Cell type*	*Age*	*Gender*	*Body area*
1	ASCs	27	female	abdomen
2	ASCs	51	female	breast
3	ASCs	71	male	unknown
4	ASCs	47	female	abdomen
5	ASCs	41	female	abdomen
6	ASCs	35	female	hips
7	ASCs	42	female	breast
8	ASCs	38	male	abdomen
9	ASCs	70	female	buttocks
10	ASCs	46	female	abdomen
11	ASCs	32	female	hips
	PBMCs	30	female	
	PBMCs	35	male	
	PBMCs	66	male	
