# Supplementary material for: Protein kinase inhibitor SU6668 attenuates positive regulation of Gli proteins in cancer and multipotent progenitor cells
Source: Biochim Biophys Acta. 2014 Apr;1843(4):703–14. doi: 10.1016/j.bbamcr.2014.01.003 (PMC3946003; doi:10.1016/j.bbamcr.2014.01.003)
Supplement: Supplementary Table 2 — List of antibodies. [file mmc2.pdf]

Supplementary Table 2 List of antibodies

Cat.no	*Antibody*	*Supplier*	*Dilution*	*Super Signal West Substrate (Thermo Scientific)*
sc-18661	A-FABP (C-15)	Santa Cruz Biotechnology	1:1000	Pico
MAB3604	Adiponectin	Chemicon	1:5000	Pico
MAB1330	Collagen type II	Millipore	1:2000	Pico
ab5097	Ctgf	Abcam	1:1000	Pico
sc-55580	FAS (A-5)	Santa Cruz Biotechnology	1:1000	Pico
G8795	Gapdh	Sigma-Aldrich	1:8000	Pico
ab7523	Gli1	Abcam	1:10000	Femto
sc-20291	Gli2 (G-20)	Santa Cruz Biotechnology	1:500	Femto
AF3635	Gli2	R&D Systems	1:1000	Dura
ab16048	Lamin B1	Abcam	1:1000	Pico
SA-372	Matrix metalloproteinase 13 (MMP13)	Biomol	1:3000	Pico
A8592	M2-FLAG-HRP	Sigma-Aldrich	1:5000	Pico
sc-30044	Osteocalcin (FL-100) (BGLAP)	Santa Cruz Biotechnology	1:500	Dura
sc-10591	OPN (K-20)	Santa Cruz Biotechnology	1:500	Dura
05-914	Smad2/3 C4T	Upstate	1:1000	Dura
AB3849	p-Smad2 (SER465/467)	Chemicon	1:2000	Pico
AB5535	Sox9	Millipore	1:2000	Dura
ab6721	HRP-conjugated goat anti-rabbit	Abcam	1:15000	-
ab6728	HRP-conjugated rabbit anti-mouse	Abcam	1:15000	-
ab6741	HRP-conjugated rabbit anti-goat	Abcam	1:15000	-
