# Supplementary material for: Protein kinase inhibitor SU6668 attenuates positive regulation of Gli proteins in cancer and multipotent progenitor cells
Source: Biochim Biophys Acta. 2014 Apr;1843(4):703–14. doi: 10.1016/j.bbamcr.2014.01.003 (PMC3946003; doi:10.1016/j.bbamcr.2014.01.003)
Supplement: Supplementary Table 3 — List of primers. [file mmc3.pdf]

Supplementary Table 3 List of primers

Gene	*Direction*	*Sequence 5'-3'*	*GenBank Accession number*	*Expected product length, nt*
GAPDH	sense<br>antisense	CTCTCTGCTCCTCCTGTTCGAC<br>TGAGCGATGTGGCTCGGCT	NM_002046.4	68
GLI1	sense<br>antisense	CAAGTGCACGTTTGAAGGGT<br>CTTCTCATTGGAATGGGTCC	NM_001167609.1<br>NM_001160045.1<br>NM_005269.2	177
	sense<br>antisense	CCTTCAGCAATGCCAGTGA<br>CTAGGATCTGTATAGCGTTT	NM_001167609.1<br>NM_001160045.1<br>NM_005269.2	108
GLI2	sense<br>antisense	CATCTCCACGACTACCTCAAC<br>ACTAGCGAGTTGGGTGAGG	NM_005270.4	157
	sense<br>antisense	GACATGCGACACCAGGAAGGAAGGT<br>GCCGGATCAAGGAGATGTCAGAGATG	NM_005270.4	114
ULK3	sense<br>antisense	AAGGAGCAGGTCAAGATGAG<br>GTGCAAGAGCTACGAACAGA	NM_001099436.1	88
	sense<br>antisense	AACGGAATATCTCTCACCTG<br>TGTGCGAAACCAAAGTCTGC	NM_001099436.1	96
PTCH1	sense<br>antisense	GAATGGATCAGACGATGGAG<br>GGATTAATGATGCCATCTGC	NM_001083605.1<br>NM_001083602.1<br>NM_001083604.1<br>NM_001083607.1<br>NM_000264.3<br>NM_001083606.1<br>NM_001083603.1	125
	sense<br>antisense	ATCCCTTTTGAGGACAGGAC<br>ATTGCAGGAAAAATGAGCAG	NM_001083605.1<br>NM_001083602.1<br>NM_001083604.1<br>NM_001083607.1<br>NM_000264.3<br>NM_001083606.1<br>NM_001083603.1	174
SHH	sense<br>antisense	ACTCCGAGCGATTTAAGGAACT<br>CAGACGTGGTGATGTCCACTG	NM_000193.2	248
SMO	sense<br>antisense	GGGAGGCTACTTCCTCATCC<br>GGCAGCTGAAGGTAATGAGC	NM_005631.4	166
CTGF	sense<br>antisense	GCTTACCGACTGGAAGACACG<br>CGGATGCACTTTTTGCCCTT	NM_001901.2	232
SNAI1	sense<br>antisense	GCCTTCAACTGCAAATACTGC<br>CTTCTTGACATCTGAGTGGGTC	NM_005985.3	248
SMAD7	sense<br>antisense	ACTCCAGATACCCGATGGATTT<br>CCTCCCAGTATGCCACCAC	NM_001190822.1<br>NM_001190821.1<br>NM_005904.3	173
PAI1	sense<br>antisense	CGATGGCCATTACTACGACA<br>GTTGGTGAGGGCAGAGAGAG	NM_000602.4	108
Hprt	sense<br>antisense	CAGTCCCAGCGTCGTGATTA<br>AGCAAGTCTTTCAGTCCTGTC	NM_013556.2	138
Gli1	sense<br>antisense	AACTCCACAGGCACACAGGAT<br>ATGCTCACTGTTGATGTGGT	NM_010296.2	173
	sense<br>antisense	ACGTTTGAAGGCTGTCGGAA<br>CACACGTATGGCTTCTCATT	NM_010296.2	181

Supplementary Table 3 List of primers (continued)

Gene	*Direction*	*Sequence 5'-3'*	*GenBank Accession number*	*Expected product length, nt*
Ptch1	sense<br>antisense	TTGTCAGATACTTCTTTGCC<br>ATTGGCTGGAGACACCTCAG	NM_008957.2	121
	sense<br>antisense	TCCTCGCTTACAAACTCCTG<br>GGATTAATGATGCCATCTGC	NM_008957.2	105
Gli2	sense<br>antisense	CGCTTTGAGCAGCTGAAGAG<br>GACAGAACCATTGACTGGAA	NM_001081125.1	119
	sense<br>antisense	GACCTGAGGAGAGTGTGGAG<br>CTCGCTGCTGCAGGATGACT	NM_001081125.1	133
Ulk3	sense<br>antisense	AGGAGCAGATCAAGATAAG<br>GTGCAAGAACTACGAACAGA	NM_027895.1	87
	sense<br>antisense	ACGAAACATCTCTCACTTG<br>TGCTGGGCAAAGCCAAAGTC	NM_027895.1	98
Runx2	sense<br>antisense	CAGAGGGCACAAGTTCTATCTG<br>CTCACGTCGCTCATCTTGC	NM_009820.4<br>NM_001146038.1	167
	sense<br>antisense	TAACTTGTGGCTGTTGTGATGCG<br>CTCACGTCGCTCATCTTGC	NM_001145920.1	103
Sp7	sense<br>antisense	GATGGCGTCCTCTCTGCTTG<br>GTCAGCGTATGGCTTCTTTGTG	NM_130458.3	150
